# Di-μ-chlorido-bis­[(2,2′-bipyridine-5,5′-dicarb­oxy­lic acid-κ^2^ *N*,*N*′)chloridocopper(II)] dimethyl­formamide tetra­solvate

**DOI:** 10.1107/S1600536812051422

**Published:** 2013-01-04

**Authors:** Sigurd Øien, David Stephen Wragg, Karl Petter Lillerud, Mats Tilset

**Affiliations:** a inGAP Centre for Research Based Innovation, Department of Chemistry, University of Oslo, 0315 Oslo, Norway

## Abstract

In the title compound, [Cu_2_Cl_4_(C_12_H_8_N_2_O_4_)_2_]·4C_3_H_7_NO, which contains a chloride-bridged centrosymmetric Cu^II^ dimer, the Cu^II^ atom is in a distorted square-pyramidal 4 + 1 coordination geometry defined by the N atoms of the chelating 2,2′-bipyridine ligand, a terminal chloride and two bridging chloride ligands. Of the two independent dimethyl­formamide mol­ecules, one is hydrogen bonded to a single –COOH group, while one links two adjacent –COOH groups *via* a strong accepted O—H⋯O and a weak donated C(O)—H⋯O hydrogen bond. Two of these last mol­ecules and the two –COOH groups form a centrosymmetric hydrogen-bonded ring in which the CH=O and the –COOH groups by disorder adopt two alternate orientations in a 0.44:0.56 ratio. These hydrogen bonds link the Cu^II^ complex mol­ecules and the dimethyl­formamide solvent mol­ecules into infinite chains along [-111]. Slipped π–π stacking inter­actions between two centrosymmetric pyridine rings (centroid–centroid distance = 3.63 Å) contribute to the coherence of the structure along [0-11].

## Related literature

For related structures with similar coordination geometry around the copper atoms, see: Goddard *et al.* (1990 ▶); Tynan *et al.* (2005 ▶); Han *et al.* (2008 ▶); Liu *et al.* (2009 ▶); Qi *et al.* (2009 ▶). For other related structures of chloro bipyridine copper complexes, see: Wang *et al.* (2004 ▶); Zhao *et al.* (2010 ▶).
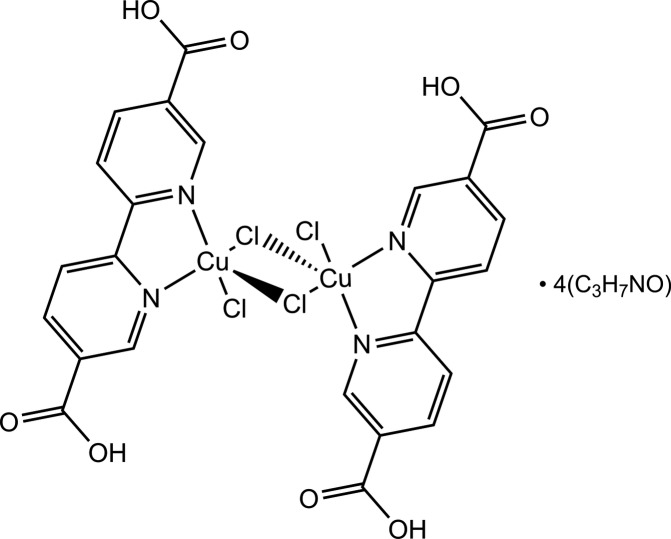


## Experimental

### 

#### Crystal data

[Cu_2_Cl_4_(C_12_H_8_N_2_O_4_)_2_]·4C_3_H_7_NO
*M* *_r_* = 1049.66
Triclinic, 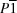

*a* = 8.917 (5) Å
*b* = 11.030 (6) Å
*c* = 12.179 (7) Å
α = 83.171 (6)°
β = 73.903 (6)°
γ = 68.332 (6)°
*V* = 1069.4 (11) Å^3^
*Z* = 1
Mo *K*α radiation
μ = 1.32 mm^−1^
*T* = 100 K
0.20 × 0.15 × 0.02 mm

#### Data collection

Bruker APEXII CCD diffractometer
Absorption correction: multi-scan (*SADABS*; Bruker, 2005 ▶) *T*_min_ = 0.789, *T*_max_ = 0.974
9231 measured reflections
4824 independent reflections
3969 reflections with *I* > 2σ(*I*)
*R*_int_ = 0.021

#### Refinement

*R*[*F* ^2^ > 2σ(*F* ^2^)] = 0.029
*wR*(*F* ^2^) = 0.068
*S* = 1.02
4824 reflections
295 parameters
H-atom parameters constrained
Δρ_max_ = 0.43 e Å^−3^
Δρ_min_ = −0.37 e Å^−3^

### 

Data collection: *APEX2* (Bruker, 2005 ▶); cell refinement: *SAINT* (Bruker, 2005 ▶); data reduction: *SAINT*; program(s) used to solve structure: *SHELXS97* (Sheldrick, 2008 ▶); program(s) used to refine structure: *SHELXL97* (Sheldrick, 2008 ▶); molecular graphics: *DIAMOND* (Brandenburg, 2006 ▶) and *Materials Studio* (Accelrys, 2010 ▶); software used to prepare material for publication: *publCIF* (Westrip, 2010 ▶).

## Supplementary Material

Crystal structure: contains datablock(s) I, global. DOI: 10.1107/S1600536812051422/qk2049sup1.cif

Structure factors: contains datablock(s) I. DOI: 10.1107/S1600536812051422/qk2049Isup2.hkl

Additional supplementary materials:  crystallographic information; 3D view; checkCIF report

## Figures and Tables

**Table 1:** Selected bond lengths (Å)

N1—Cu1	2.0337 (18)
N2—Cu1	2.0361 (17)
Cl1—Cu1	2.2525 (10)
Cl2—Cu1^i^	2.2804 (10)
Cl2—Cu1	2.7183 (12)

Symmetry code: (i) 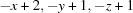.

**Table 2:** Hydrogen-bond geometry (Å, °)

*D*—H⋯*A*	*D*—H	H⋯*A*	*D*⋯*A*	*D*—H⋯*A*
O2—H2⋯O6*B* ^ii^	0.82	1.72	2.515 (3)	161
O1—H1⋯O6*A* ^iii^	0.82	1.75	2.536 (4)	161
O3—H3⋯O5	0.82	1.72	2.541 (2)	177
C21*A*—H21*A*⋯O2^iv^	0.93	2.71	3.591 (3)	158
C21*B*—H21*B*⋯O1^iii^	0.93	2.72	3.603 (3)	159

Symmetry codes: (ii) 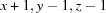; (iii) 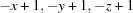; (iv) 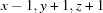.

**Fig. 1.**
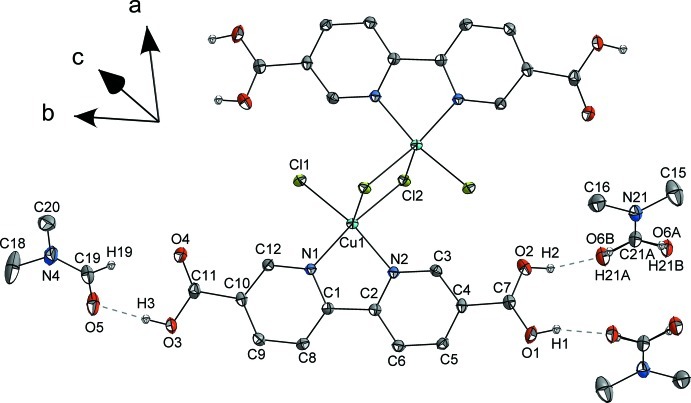
The molecular structure of (I), with atom labels and 50% probability displacement ellipsoids for non-H atoms showing also the disorder of one COOH group (C7 etc.) and one DMF molecule (C21A etc).

**Fig. 2.**
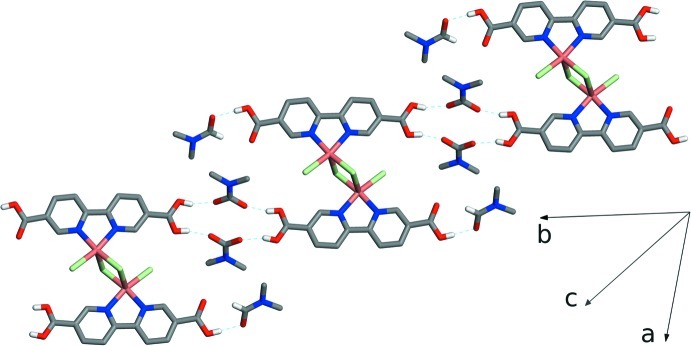
The packing of (I), showing the hydrogen bonded chains. Hydrogen atoms (except amide and carboxylic) are omitted and hydrogen bonds are shown as dashed lines.

**Fig. 3.**
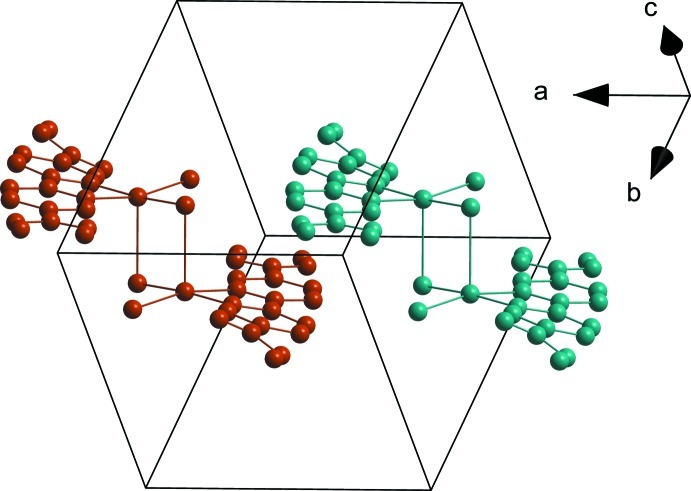
Slipped π-π stacking interaction between the pyridine rings 1 (N1–C1–C8–C9–C10–C12) of two neighboring Cu complexes related by inversion (I).

## supplementary crystallographic information

### Comment

In recent years, linear dicarboxylic acids have attracted much attention for their usage as linkers in metal-organic frameworks. This diverse class of porous materials can be utilized as heterogeneous catalysts or selective adsorbents, by incorporating active species onto the linkers. The reported compound (Fig. 1) consists of centrosymmetric dinuclear Cu complexes hydrogen bonded to four DMF molecules *via* O—H···O and C—H···O links. These Cu dimers and the DMF molecules create hydrogen bonded chains parallell to [111] (Fig. 2). The copper atoms have a slightly distorted square-pyramidal coordination by two N and three Cl atoms (two short and one long Cu—Cl bonds;Table 1), as observed in similar copper dimer complexes reported for instance by Goddard *et al.* (1990),Tynan *et al.* (2005), Han *et al.* (2008), Liu *et al.* (2009) and Qi *et al.* (2009). Fig. 1 and Fig. 2 show that the COOH group of O1–C7–O2 and the second DMF molecule (C21, O6A/O6B, N21, C15, C16) form a centrosymmetric hydrogen bond ring with alternating strong O—H···O(DMF) and weak C(DMF)—H···O hydrogen bonds. Due to a synchronous orientation disorder of the COOH groups and the DMF molecules the hydrogen bonds in these rings can adopt a clock or an anticlockwise sense in 0.44/0.56 ratio. Consequently, the observed bond distances C7—O1 = 1.261 (3) Å and C7—O2 = 1.264 (3) Å are approximately an average of the single and double bond distances of an ordered COOH group (e.g. C11═O4 = 1.209 (3) and C11—O3 = 1.311 (3) Å in the title compound). Apart from hydrogen bonding the structure of the title compound is held together by slipped π-π stacking interactions between centrosymmetric pairs of pyridine ring 1 (N1–C1–C8–C9–C10–C12). They show stacking distances of ca. 3.33 Å which are effective along [011] (Fig. 3). A polymeric copper(II) complex with the same organoligand as in (I) but with a Cu/Cl ratio of 1:1 has been reported by Zhao *et al.* (2010).

### Experimental

5,5'-dimethyl-2,2'-bipyridine was purchased from Sigma-Aldrich and oxidized with K_2_Cr_2_O_7_ to 2,2'-bipyridine-5,5'-dicarboxylic acid according to literature methods. CuCl_2_.2H_2_O (>99%, Sigma-Aldrich) and dimethylformamide (DMF) (>99.5%, Merck) were used as received. 100 mg (0.41 mmol) H_2_bpydc was dissolved in 10 ml of water, using a minimal amount of KOH. 70 mg (0.41 mmol) CuCl_2_.2H_2_O was dissolved in water. When the two solutions were combined, a blue precipitate was immediately formed. Dilute HCl was added until pH was 4. The blue microcrystalline precipitate (96 mg) was recovered, dried and dissolved in 5 ml of DMF along with 50 µ*L* conc. HCl, giving a green solution. 1 ml of the solution was transferred to a small vial. The crystals were precipitated by vapor diffusion, using water as antisolvent.

### Refinement

All H atoms were placed in geometrically idealized positions, with C_sp2_—H = 0.93 Å, C_sp3_—H = 0.96 Å, O—H = 0.82 Å and constrained to ride on their parent atoms, with *U*_iso_(H) = 1.2*U*_eq_(C_sp2_) or 1.5*U*_eq_(C_sp3_,O). The atoms O6 A/B, C21 A/B and H21 A/B of one DMF molecule and H1/2 of a COOH group are disordered over 2 sites with refined occupancies of 0.437 (4) (part A and H1) and 0.563 (4) (part B and H2).

### Crystal data

**Table:**

[Cu_2_Cl_4_(C_12_H_8_N_2_O_4_)_2_]·4C_3_H_7_NO	*Z* = 1
*M**_r_* = 1049.66	*F*(000) = 538
Triclinic, *P*1	*D*_x_ = 1.627 Mg m^−^^3^
Hall symbol: -P 1	Mo *K*α radiation, λ = 0.71073 Å
*a* = 8.917 (5) Å	Cell parameters from 3373 reflections
*b* = 11.030 (6) Å	θ = 2.5–27.4°
*c* = 12.179 (7) Å	µ = 1.32 mm^−^^1^
α = 83.171 (6)°	*T* = 100 K
β = 73.903 (6)°	Prism, green
γ = 68.332 (6)°	0.20 × 0.15 × 0.02 mm
*V* = 1069.4 (11) Å^3^	

### Data collection

**Table:**

Bruker APEXII CCD diffractometer	4824 independent reflections
Radiation source: fine-focus sealed tube	3969 reflections with *I* > 2σ(*I*)
Graphite monochromator	*R*_int_ = 0.021
φ and ω scans	θ_max_ = 27.9°, θ_min_ = 1.7°
Absorption correction: multi-scan (*SADABS*; Bruker, 2005)	*h* = −11→11
*T*_min_ = 0.789, *T*_max_ = 0.974	*k* = −14→14
9231 measured reflections	*l* = −15→15

### Refinement

**Table:**

Refinement on *F*^2^	Primary atom site location: structure-invariant direct methods
Least-squares matrix: full	Secondary atom site location: difference Fourier map
*R*[*F*^2^ > 2σ(*F*^2^)] = 0.029	Hydrogen site location: inferred from neighbouring sites
*wR*(*F*^2^) = 0.068	H-atom parameters constrained
*S* = 1.02	*w* = 1/[σ^2^(*F*_o_^2^) + (0.0259*P*)^2^ + 0.590*P*] where *P* = (*F*_o_^2^ + 2*F*_c_^2^)/3
4824 reflections	(Δ/σ)_max_ = 0.001
295 parameters	Δρ_max_ = 0.43 e Å^−^^3^
0 restraints	Δρ_min_ = −0.37 e Å^−^^3^

### Special details

**Table:**

Geometry. All e.s.d.'s (except the e.s.d. in the dihedral angle between two l.s. planes) are estimated using the full covariance matrix. The cell e.s.d.'s are taken into account individually in the estimation of e.s.d.'s in distances, angles and torsion angles; correlations between e.s.d.'s in cell parameters are only used when they are defined by crystal symmetry. An approximate (isotropic) treatment of cell e.s.d.'s is used for estimating e.s.d.'s involving l.s. planes.
Refinement. Refinement of *F*^2^ against ALL reflections. The weighted *R*-factor *wR* and goodness of fit *S* are based on *F*^2^, conventional *R*-factors *R* are based on *F*, with *F* set to zero for negative *F*^2^. The threshold expression of *F*^2^ > σ(*F*^2^) is used only for calculating *R*-factors(gt) *etc*. and is not relevant to the choice of reflections for refinement. *R*-factors based on *F*^2^ are statistically about twice as large as those based on *F*, and *R*- factors based on ALL data will be even larger.

### Fractional atomic coordinates and isotropic or equivalent isotropic displacement parameters (Å^2^)

**Table:**

	*x*	*y*	*z*	*U*_iso_*/*U*_eq_	Occ. (<1)
C1	0.5061 (2)	0.75714 (18)	0.49804 (17)	0.0142 (4)	
C2	0.5665 (2)	0.64520 (18)	0.42163 (16)	0.0141 (4)	
C3	0.7981 (2)	0.47716 (18)	0.32901 (16)	0.0150 (4)	
H3A	0.9128	0.4329	0.3112	0.018*	
C4	0.6997 (3)	0.43285 (18)	0.28504 (17)	0.0157 (4)	
C5	0.5292 (2)	0.49803 (19)	0.31204 (17)	0.0168 (4)	
H5	0.4611	0.4698	0.2838	0.020*	
C6	0.4604 (3)	0.60601 (19)	0.38163 (17)	0.0170 (4)	
H6	0.3459	0.6511	0.4010	0.020*	
C7	0.7786 (3)	0.31742 (19)	0.20877 (17)	0.0174 (4)	
C8	0.3416 (3)	0.84090 (19)	0.52720 (17)	0.0175 (4)	
H8	0.2620	0.8292	0.4980	0.021*	
C9	0.2973 (3)	0.94225 (19)	0.60049 (18)	0.0178 (4)	
H9	0.1884	1.0014	0.6192	0.021*	
C10	0.4173 (3)	0.95414 (18)	0.64538 (17)	0.0161 (4)	
C11	0.3799 (3)	1.0583 (2)	0.72813 (18)	0.0202 (4)	
C12	0.5801 (3)	0.86824 (19)	0.61159 (17)	0.0164 (4)	
H12	0.6608	0.8774	0.6412	0.020*	
C15	0.3041 (4)	0.7442 (2)	1.1227 (2)	0.0452 (7)	
H15A	0.3318	0.7211	1.0441	0.068*	
H15B	0.2396	0.6952	1.1693	0.068*	
H15C	0.4047	0.7248	1.1465	0.068*	
C16	0.1570 (3)	0.9324 (3)	1.2499 (2)	0.0327 (6)	
H16A	0.0844	1.0220	1.2507	0.049*	
H16B	0.2539	0.9263	1.2734	0.049*	
H16C	0.0992	0.8818	1.3016	0.049*	
C18	0.2042 (5)	1.4697 (3)	1.0417 (2)	0.0590 (10)	
H18A	0.1608	1.5052	0.9764	0.088*	
H18B	0.2955	1.4971	1.0395	0.088*	
H18C	0.1180	1.5003	1.1103	0.088*	
C19	0.2560 (3)	1.2678 (2)	0.95630 (19)	0.0272 (5)	
H19	0.2955	1.1771	0.9602	0.033*	
C20	0.3184 (3)	1.2590 (3)	1.1386 (2)	0.0434 (7)	
H20A	0.3579	1.1668	1.1265	0.065*	
H20B	0.2275	1.2811	1.2059	0.065*	
H20C	0.4073	1.2827	1.1483	0.065*	
C21A	0.1675 (3)	0.9574 (2)	1.04753 (19)	0.0242 (5)	0.437 (4)
H21A	0.1017	1.0445	1.0620	0.029*	0.437 (4)
O6A	0.2100 (5)	0.9214 (3)	0.9473 (3)	0.0255 (11)	0.437 (4)
C21B	0.1675 (3)	0.9574 (2)	1.04753 (19)	0.0242 (5)	0.563 (4)
H21B	0.1983	0.9165	0.9781	0.029*	0.563 (4)
O6B	0.0912 (4)	1.0793 (3)	1.0485 (2)	0.0304 (9)	0.563 (4)
N1	0.6254 (2)	0.77252 (15)	0.53787 (14)	0.0139 (3)	
N2	0.7337 (2)	0.58081 (15)	0.39584 (14)	0.0140 (3)	
N4	0.2618 (3)	1.32944 (19)	1.04017 (16)	0.0293 (4)	
N21	0.2077 (2)	0.88264 (17)	1.13500 (15)	0.0227 (4)	
O1	0.6831 (2)	0.27950 (15)	0.17378 (14)	0.0278 (4)	
H1	0.7387	0.2161	0.1329	0.042*	0.437 (4)
O2	0.93592 (19)	0.26688 (15)	0.18333 (14)	0.0293 (4)	
H2	0.9662	0.2047	0.1415	0.044*	0.563 (4)
O3	0.23516 (19)	1.15187 (14)	0.73405 (13)	0.0233 (3)	
H3	0.2215	1.2066	0.7795	0.035*	
O4	0.4777 (2)	1.05397 (17)	0.78151 (15)	0.0347 (4)	
O5	0.2021 (2)	1.32076 (15)	0.87227 (13)	0.0337 (4)	
Cl1	0.97529 (6)	0.78040 (5)	0.52466 (4)	0.01868 (11)	
Cl2	0.88428 (6)	0.47339 (5)	0.63849 (4)	0.01680 (11)	
Cu1	0.86273 (3)	0.65512 (2)	0.46635 (2)	0.01421 (7)	

### Atomic displacement parameters (Å^2^)

**Table:**

	*U*^11^	*U*^22^	*U*^33^	*U*^12^	*U*^13^	*U*^23^
C1	0.0145 (10)	0.0134 (9)	0.0158 (10)	−0.0059 (8)	−0.0049 (8)	0.0017 (7)
C2	0.0139 (10)	0.0138 (9)	0.0148 (10)	−0.0054 (8)	−0.0038 (8)	0.0010 (7)
C3	0.0136 (10)	0.0149 (9)	0.0153 (10)	−0.0035 (8)	−0.0042 (8)	0.0005 (8)
C4	0.0191 (10)	0.0127 (9)	0.0153 (10)	−0.0062 (8)	−0.0034 (8)	−0.0001 (7)
C5	0.0177 (10)	0.0170 (10)	0.0187 (10)	−0.0082 (8)	−0.0067 (8)	−0.0007 (8)
C6	0.0137 (10)	0.0173 (10)	0.0199 (11)	−0.0050 (8)	−0.0050 (8)	0.0000 (8)
C7	0.0195 (11)	0.0152 (9)	0.0159 (10)	−0.0055 (8)	−0.0030 (8)	−0.0006 (8)
C8	0.0151 (10)	0.0181 (10)	0.0209 (11)	−0.0062 (8)	−0.0064 (8)	−0.0013 (8)
C9	0.0139 (10)	0.0158 (10)	0.0207 (11)	−0.0031 (8)	−0.0027 (8)	−0.0011 (8)
C10	0.0180 (10)	0.0138 (9)	0.0151 (10)	−0.0054 (8)	−0.0023 (8)	0.0000 (8)
C11	0.0207 (11)	0.0207 (10)	0.0176 (11)	−0.0079 (9)	0.0002 (9)	−0.0046 (8)
C12	0.0167 (10)	0.0174 (10)	0.0164 (10)	−0.0071 (8)	−0.0042 (8)	−0.0018 (8)
C15	0.0600 (19)	0.0260 (13)	0.0336 (15)	−0.0043 (13)	−0.0032 (14)	0.0031 (11)
C16	0.0285 (13)	0.0451 (15)	0.0223 (12)	−0.0123 (11)	−0.0022 (10)	−0.0053 (11)
C18	0.116 (3)	0.0361 (16)	0.0334 (16)	−0.0372 (18)	−0.0143 (18)	−0.0073 (13)
C19	0.0318 (13)	0.0209 (11)	0.0236 (12)	−0.0049 (10)	−0.0021 (10)	−0.0068 (9)
C20	0.0328 (15)	0.0567 (18)	0.0327 (15)	0.0025 (13)	−0.0144 (12)	−0.0170 (13)
C21A	0.0257 (12)	0.0203 (11)	0.0270 (12)	−0.0070 (9)	−0.0061 (10)	−0.0068 (9)
O6A	0.035 (2)	0.0194 (19)	0.020 (2)	−0.0058 (16)	−0.0081 (16)	−0.0050 (14)
C21B	0.0257 (12)	0.0203 (11)	0.0270 (12)	−0.0070 (9)	−0.0061 (10)	−0.0068 (9)
O6B	0.0358 (18)	0.0216 (15)	0.0283 (17)	−0.0037 (13)	−0.0062 (13)	−0.0056 (12)
N1	0.0125 (8)	0.0136 (8)	0.0165 (8)	−0.0046 (6)	−0.0047 (7)	−0.0003 (6)
N2	0.0132 (8)	0.0138 (8)	0.0152 (8)	−0.0047 (7)	−0.0040 (7)	−0.0001 (6)
N4	0.0333 (12)	0.0327 (11)	0.0208 (10)	−0.0114 (9)	−0.0017 (9)	−0.0097 (8)
N21	0.0223 (10)	0.0221 (9)	0.0213 (10)	−0.0068 (8)	−0.0020 (8)	−0.0032 (7)
O1	0.0317 (9)	0.0262 (8)	0.0302 (9)	−0.0137 (7)	−0.0056 (7)	−0.0114 (7)
O2	0.0210 (9)	0.0259 (8)	0.0344 (9)	0.0000 (7)	−0.0033 (7)	−0.0114 (7)
O3	0.0269 (9)	0.0170 (7)	0.0219 (8)	−0.0014 (6)	−0.0053 (7)	−0.0074 (6)
O4	0.0239 (9)	0.0420 (10)	0.0379 (10)	−0.0025 (8)	−0.0100 (8)	−0.0239 (8)
O5	0.0581 (12)	0.0210 (8)	0.0189 (8)	−0.0089 (8)	−0.0109 (8)	−0.0028 (7)
Cl1	0.0153 (2)	0.0184 (2)	0.0249 (3)	−0.00683 (19)	−0.0069 (2)	−0.00293 (19)
Cl2	0.0120 (2)	0.0202 (2)	0.0173 (2)	−0.00488 (19)	−0.00233 (18)	−0.00277 (19)
Cu1	0.01079 (13)	0.01549 (12)	0.01676 (13)	−0.00388 (9)	−0.00401 (9)	−0.00304 (9)

### Geometric parameters (Å, º)

**Table:**

C1—N1	1.355 (3)	C15—H15C	0.9600
C1—C8	1.386 (3)	C16—N21	1.453 (3)
C1—C2	1.478 (3)	C16—H16A	0.9600
C2—N2	1.355 (3)	C16—H16B	0.9600
C2—C6	1.388 (3)	C16—H16C	0.9600
C3—N2	1.333 (3)	C18—N4	1.439 (3)
C3—C4	1.391 (3)	C18—H18A	0.9600
C3—H3A	0.9300	C18—H18B	0.9600
C4—C5	1.382 (3)	C18—H18C	0.9600
C4—C7	1.495 (3)	C19—O5	1.242 (3)
C5—C6	1.388 (3)	C19—N4	1.315 (3)
C5—H5	0.9300	C19—H19	0.9300
C6—H6	0.9300	C20—N4	1.456 (3)
C7—O1	1.261 (3)	C20—H20A	0.9600
C7—O2	1.264 (3)	C20—H20B	0.9600
C8—C9	1.385 (3)	C20—H20C	0.9600
C8—H8	0.9300	C21A—O6A	1.241 (4)
C9—C10	1.381 (3)	C21A—N21	1.315 (3)
C9—H9	0.9300	C21A—H21A	0.9300
C10—C12	1.386 (3)	N1—Cu1	2.0337 (18)
C10—C11	1.501 (3)	N2—Cu1	2.0361 (17)
C11—O4	1.209 (3)	O1—H1	0.8200
C11—O3	1.311 (3)	O2—H2	0.8200
C12—N1	1.339 (3)	O3—H3	0.8200
C12—H12	0.9300	Cl1—Cu1	2.2525 (10)
C15—N21	1.451 (3)	Cl2—Cu1^i^	2.2804 (10)
C15—H15A	0.9600	Cl2—Cu1	2.7183 (12)
C15—H15B	0.9600		
			
N1—C1—C8	121.94 (18)	H16A—C16—H16C	109.5
N1—C1—C2	114.49 (17)	H16B—C16—H16C	109.5
C8—C1—C2	123.57 (18)	N4—C18—H18A	109.5
N2—C2—C6	122.18 (18)	N4—C18—H18B	109.5
N2—C2—C1	114.96 (16)	H18A—C18—H18B	109.5
C6—C2—C1	122.83 (18)	N4—C18—H18C	109.5
N2—C3—C4	122.29 (19)	H18A—C18—H18C	109.5
N2—C3—H3A	118.9	H18B—C18—H18C	109.5
C4—C3—H3A	118.9	O5—C19—N4	125.3 (2)
C5—C4—C3	118.91 (18)	O5—C19—H19	117.3
C5—C4—C7	120.96 (18)	N4—C19—H19	117.3
C3—C4—C7	120.13 (18)	N4—C20—H20A	109.5
C4—C5—C6	119.42 (18)	N4—C20—H20B	109.5
C4—C5—H5	120.3	H20A—C20—H20B	109.5
C6—C5—H5	120.3	N4—C20—H20C	109.5
C5—C6—C2	118.45 (19)	H20A—C20—H20C	109.5
C5—C6—H6	120.8	H20B—C20—H20C	109.5
C2—C6—H6	120.8	O6A—C21A—N21	125.4 (3)
O1—C7—O2	125.27 (19)	O6A—C21A—H21A	117.3
O1—C7—C4	117.40 (18)	N21—C21A—H21A	117.3
O2—C7—C4	117.32 (18)	C12—N1—C1	118.42 (17)
C9—C8—C1	119.05 (19)	C12—N1—Cu1	126.23 (14)
C9—C8—H8	120.5	C1—N1—Cu1	115.10 (13)
C1—C8—H8	120.5	C3—N2—C2	118.75 (17)
C10—C9—C8	118.98 (19)	C3—N2—Cu1	126.29 (14)
C10—C9—H9	120.5	C2—N2—Cu1	114.96 (13)
C8—C9—H9	120.5	C19—N4—C18	121.5 (2)
C9—C10—C12	119.08 (18)	C19—N4—C20	121.4 (2)
C9—C10—C11	122.74 (18)	C18—N4—C20	117.0 (2)
C12—C10—C11	118.17 (18)	C21A—N21—C15	121.8 (2)
O4—C11—O3	124.97 (19)	C21A—N21—C16	122.4 (2)
O4—C11—C10	121.63 (19)	C15—N21—C16	115.8 (2)
O3—C11—C10	113.39 (18)	C7—O1—H1	109.5
N1—C12—C10	122.42 (18)	C7—O2—H2	109.5
N1—C12—H12	118.8	C11—O3—H3	109.5
C10—C12—H12	118.8	Cu1^i^—Cl2—Cu1	90.20 (4)
N21—C15—H15A	109.5	N1—Cu1—N2	79.91 (8)
N21—C15—H15B	109.5	N1—Cu1—Cl1	92.97 (6)
H15A—C15—H15B	109.5	N2—Cu1—Cl1	166.75 (5)
N21—C15—H15C	109.5	N1—Cu1—Cl2^i^	171.33 (5)
H15A—C15—H15C	109.5	N2—Cu1—Cl2^i^	93.45 (7)
H15B—C15—H15C	109.5	Cl1—Cu1—Cl2^i^	92.41 (5)
N21—C16—H16A	109.5	N1—Cu1—Cl2	96.09 (5)
N21—C16—H16B	109.5	N2—Cu1—Cl2	93.10 (6)
H16A—C16—H16B	109.5	Cl1—Cu1—Cl2	98.80 (4)
N21—C16—H16C	109.5	Cl2^i^—Cu1—Cl2	89.80 (4)
			
N1—C1—C2—N2	−4.8 (2)	C8—C1—N1—Cu1	−171.55 (15)
C8—C1—C2—N2	174.95 (18)	C2—C1—N1—Cu1	8.2 (2)
N1—C1—C2—C6	173.40 (18)	C4—C3—N2—C2	−0.1 (3)
C8—C1—C2—C6	−6.9 (3)	C4—C3—N2—Cu1	179.64 (14)
N2—C3—C4—C5	−0.4 (3)	C6—C2—N2—C3	0.6 (3)
N2—C3—C4—C7	178.92 (17)	C1—C2—N2—C3	178.81 (16)
C3—C4—C5—C6	0.3 (3)	C6—C2—N2—Cu1	−179.13 (15)
C7—C4—C5—C6	−179.00 (18)	C1—C2—N2—Cu1	−0.9 (2)
C4—C5—C6—C2	0.2 (3)	O5—C19—N4—C18	−0.3 (4)
N2—C2—C6—C5	−0.7 (3)	O5—C19—N4—C20	176.4 (2)
C1—C2—C6—C5	−178.74 (18)	O6A—C21A—N21—C15	−2.5 (4)
C5—C4—C7—O1	−2.5 (3)	O6A—C21A—N21—C16	178.3 (3)
C3—C4—C7—O1	178.22 (18)	C12—N1—Cu1—N2	179.08 (17)
C5—C4—C7—O2	176.55 (19)	C1—N1—Cu1—N2	−6.78 (13)
C3—C4—C7—O2	−2.7 (3)	C12—N1—Cu1—Cl1	−12.18 (16)
N1—C1—C8—C9	−0.9 (3)	C1—N1—Cu1—Cl1	161.96 (13)
C2—C1—C8—C9	179.35 (18)	C12—N1—Cu1—Cl2	87.00 (16)
C1—C8—C9—C10	−2.2 (3)	C1—N1—Cu1—Cl2	−98.86 (14)
C8—C9—C10—C12	3.1 (3)	C3—N2—Cu1—N1	−175.67 (17)
C8—C9—C10—C11	−178.12 (19)	C2—N2—Cu1—N1	4.05 (13)
C9—C10—C11—O4	166.9 (2)	C3—N2—Cu1—Cl1	126.0 (2)
C12—C10—C11—O4	−14.3 (3)	C2—N2—Cu1—Cl1	−54.2 (3)
C9—C10—C11—O3	−14.0 (3)	C3—N2—Cu1—Cl2^i^	9.96 (16)
C12—C10—C11—O3	164.78 (18)	C2—N2—Cu1—Cl2^i^	−170.32 (13)
C9—C10—C12—N1	−0.9 (3)	C3—N2—Cu1—Cl2	−80.03 (16)
C11—C10—C12—N1	−179.79 (18)	C2—N2—Cu1—Cl2	99.69 (13)
C10—C12—N1—C1	−2.2 (3)	Cu1^i^—Cl2—Cu1—N1	173.62 (5)
C10—C12—N1—Cu1	171.82 (14)	Cu1^i^—Cl2—Cu1—N2	93.44 (6)
C8—C1—N1—C12	3.1 (3)	Cu1^i^—Cl2—Cu1—Cl1	−92.40 (5)
C2—C1—N1—C12	−177.18 (16)	Cu1^i^—Cl2—Cu1—Cl2^i^	0.0

Symmetry code: (i) −*x*+2, −*y*+1, −*z*+1.

### Hydrogen-bond geometry (Å, º)

**Table:**

*D*—H···*A*	*D*—H	H···*A*	*D*···*A*	*D*—H···*A*
O2—H2···O6*B*^ii^	0.82	1.72	2.515 (3)	161
O1—H1···O6*A*^iii^	0.82	1.75	2.536 (4)	161
O3—H3···O5	0.82	1.72	2.541 (2)	177
C21*A*—H21*A*···O2^iv^	0.93	2.71	3.591 (3)	158
C21*B*—H21*B*···O1^iii^	0.93	2.72	3.603 (3)	159

Symmetry codes: (ii) *x*+1, *y*−1, *z*−1; (iii) −*x*+1, −*y*+1, −*z*+1; (iv) *x*−1, *y*+1, *z*+1.
